# Near-Fatal Aspiration of Drug Contraband

**DOI:** 10.7759/cureus.74785

**Published:** 2024-11-29

**Authors:** Don M Benson

**Affiliations:** 1 Emergency Medicine, Garden City Hospital, Michigan State University, Garden City, USA

**Keywords:** airway emergency, airway management, foreign body airway obstruction, foreign body aspiration, pulmonary aspiration

## Abstract

This case report describes a near-fatal airway obstruction in a woman attempting to conceal drug contraband in her mouth. A patient was brought to the emergency department by emergency medical services accompanied by police after she had refused to open her mouth after being detained. The patient was initially seen at triage, being awake and responsive, but still refusing to open her mouth. While in the emergency department, she rapidly deteriorated exhibiting signs of upper airway obstruction. The patient had aspirated a plastic bag presumably containing “crack” cocaine which completely occluded her trachea. Immediate intervention consisting of direct laryngoscopy and forceps removal of the foreign body was necessary to preserve life. The case is presented as a warning to law enforcement, emergency physicians, and other first responders that a patient who will not open their mouth while attempting to conceal narcotics is at risk for aspiration, airway obstruction, and death. Patients presenting to the emergency department with this history should be closely monitored in a high-acuity area with anticipation of the need for emergency intervention to achieve and maintain airway patency.

## Introduction

Foreign body aspiration (FBA) can be a life-threatening emergency that may require immediate intervention to preserve life. The National Safety Council reports that for the general population choking remains the 4th leading cause of unintentional injury and death in the United States as of 2022. During that year, there were a total of 5553 deaths (1.7 deaths/100,000) from unintentional inhalation of food or other objects which resulted in airway obstruction. In the USA, deaths from choking are increasing yearly and exceed the combined deaths from drowning, as well as fire, flames, or smoke [1]. Roughly 75% of fatalities from FBA are reported in children, and death is usually the result of hypoxic-ischemic brain injury [2,3]. Most FBA in adults is due to inhalation of organic material such as food and is slightly more common in males [4]. Aspiration of drug contraband in attempts to conceal it from law enforcement represents an unusual and potentially unrecognized cause of aspiration [5]. We report the case of a woman who, to conceal a plastic bag containing presumably “crack” cocaine from law enforcement, aspirated it and it completely occluded her airway.

## Case presentation

A 54-year-old female was brought to the emergency department (ED) by the police after being found in a motor vehicle, apparently acting strangely. The police officers reported that upon arrival at the scene, they noted her car was parked with the engine off and that the woman’s legs were protruding from the driver’s side window. As they approached her, they thought she was initially unresponsive. However, as they tried to arouse her, she attempted to leave the scene by starting the car. Offices were able to retrieve the keys from the ignition. They saw drug paraphernalia on the passenger’s seat, which supported their concern that the woman was intoxicated. During their attempts to interrogate her, the police noted that she was either impaired or feigning impairment, as she refused to open her mouth. The officers took her into custody and notified emergency medical services (EMS), who responded to the scene and attempted to evaluate her. The woman did not cooperate, continuing to refuse to open her mouth, and was transported by ambulance to the ED of a private suburban hospital via EMS for booking clearance while remaining in police custody.

Upon arrival at the ED, she was immediately evaluated while on the pre-hospital gurney in triage. The patient was sitting upright, awake, nonverbal, and refused to respond to questions or open her mouth. She appeared disheveled and looked older than her stated age. She did not seem to be in distress. A cursory physical examination did not reveal any abnormal findings.

**Figure 1:**
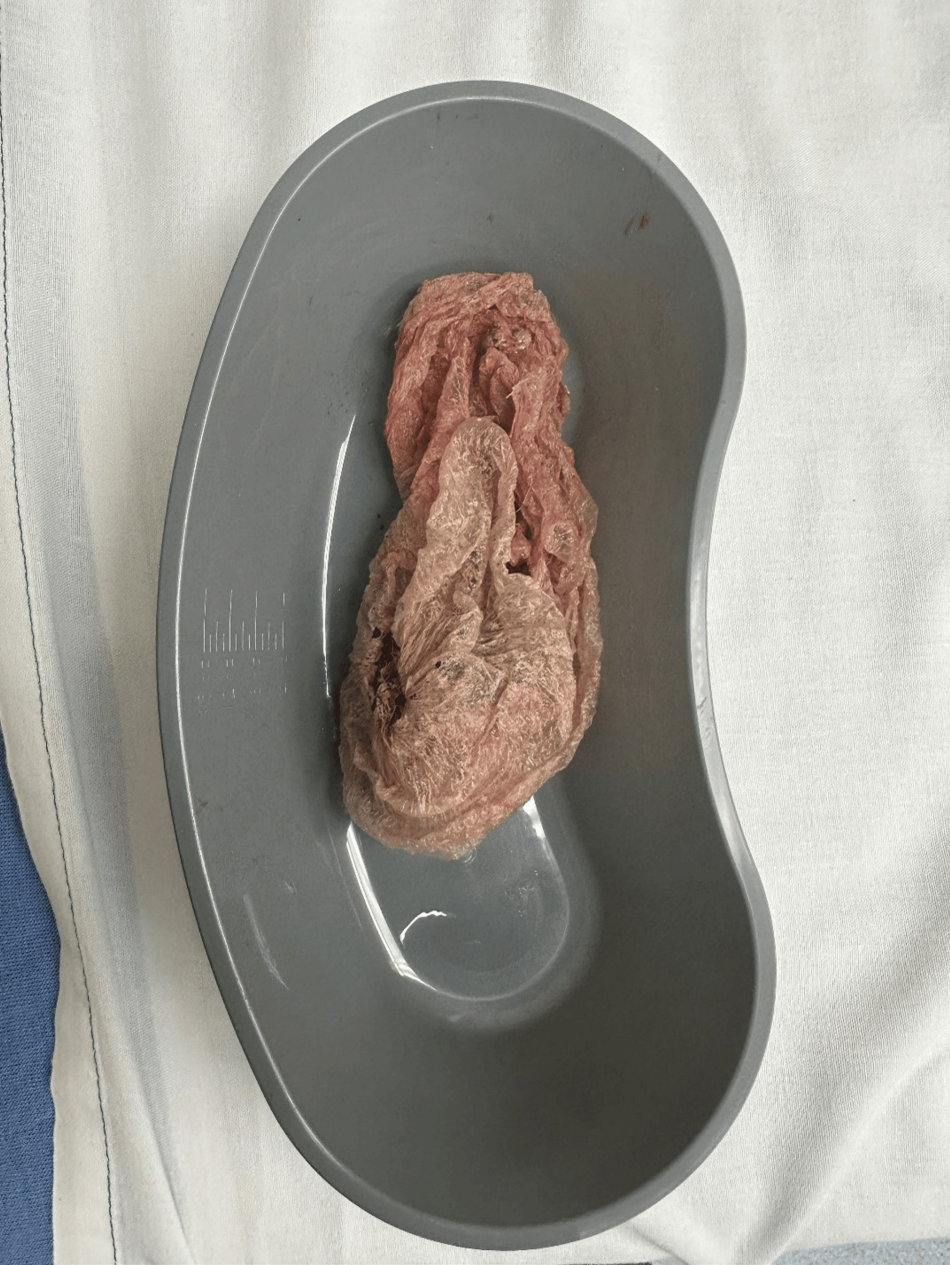
Plastic bag containing “crack” cocaine and residue which was removed from the patient’s trachea.

After the initial evaluation, the patient was assigned to an ED patient care bed. Prior to being placed on the stretcher, it was noted that she began to struggle to breathe. The patient became acutely distressed and cyanotic and exhibited market tugging of the trachea. She was moved to a high-acuity area, and placed on a stretcher where she subsequently lost consciousness. Immediate attempts to ventilate the patient with a bag/mask ventilator (BMV) were unsuccessful. A Heimlich maneuver was attempted while the patient was supine, and a repeated attempt to ventilate her with the BMV remained unsuccessful. It was noted at the time that her oxygen saturation had fallen to 27% by pulse oximetry. Direct laryngoscopy was performed using a #4 Miller laryngoscope blade, the glottis was visualized, and a plastic, pink-tinged foreign object was noted in the trachea situated below the vocal cords. The object was removed using Magill forceps, and the patient was ventilated using the BMV until acceptable oxygen saturation was achieved, then intubated with a 7.5 mm internal diameter cuffed endotracheal tube. The patient was then placed on mechanical ventilation with the following ventilator settings: FiO_2_ 100%, tidal volume 400 mL, respiratory rate 16/minute, PEEP 5 cm H_2_O, but despite this, the oxygen saturation remained at 90%. Auscultation of breast sounds post-intubation revealed diminished to absent sounds on the upper right chest. Inspection of the foreign body removed from the trachea revealed it to be a small plastic bag containing several pieces (“rocks”) of “crack” cocaine (Figure 1).

**Figure 2:**
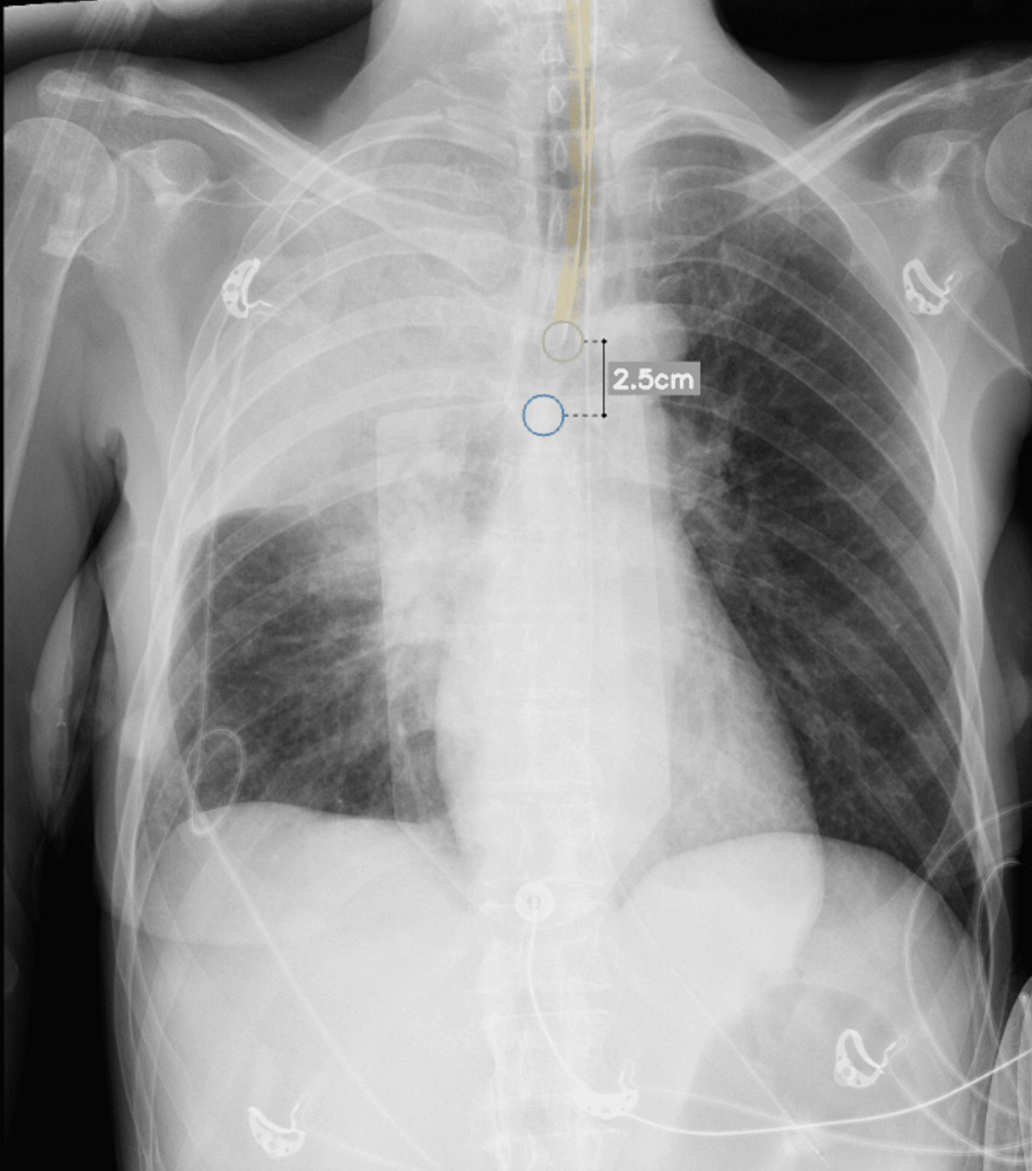
Chest radiograph obtained immediately after endotracheal intubation (pre-bronchoscopy) revealing consolidation/complete collapse of the right upper lobe.

A post-intubation radiograph was obtained, which revealed consolidation of the right upper lung with elevation of the right hemidiaphragm consistent with post-obstructive lung collapse due to FBA (Figure 2). Pulmonary/Critical Care was consulted, and a bronchoscopy was performed emergently in the ED. Several square foreign bodies (“rocks”) of “crack” cocaine measuring 4 to 5mm were retrieved by the endoscopist from the right upper and middle lobe bronchi with immediate improvement of oxygenation. The patient was then admitted to the intensive care unit (ICU). Laboratory testing revealed a normal urinalysis and urine drug screen for drugs of abuse was positive for cocaine only. Complete blood count, serum electrolytes, creatinine, blood urea nitrogen, and glucose were within normal range. Arterial blood gasses were obtained after 15 minutes of mechanical ventilation and were also within normal limits.

**Figure 3:**
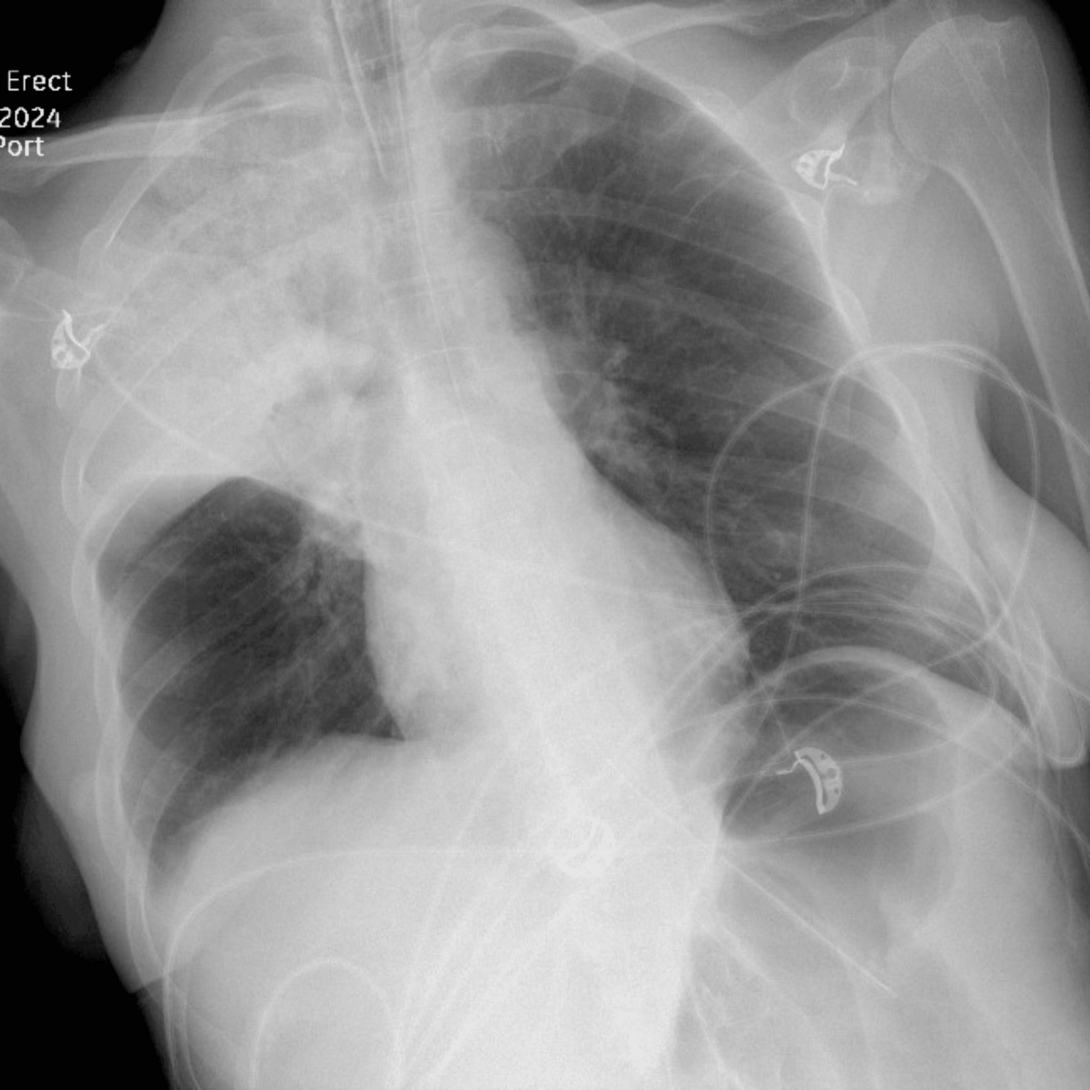
Radiograph of the chest obtained post-bronchoscopy showing some resolution of apical consolidation.

A chest radiograph was repeated post-bronchoscopy showing partial resolution of the right upper lobe collapse (Figure 3). Computed tomography (CT) of the abdomen and pelvis showed a complete collapse of the right upper lobe, with consolidations in the left upper lobe, and right middle and lower lobes. CT of the head was negative for the acute intracranial process. Gastric lavage was done on admission to the ICU to exclude swallowed material which did not reveal particulate matter. Management consisted of continued mechanical ventilation, and sedation with dexmedetomidine, fentanyl, and midazolam. Intravenous methylprednisolone was given, and antibiotics were administered for prophylaxis of aspiration pneumonia (piperacillin/tazobactam and vancomycin).

**Figure 4:**
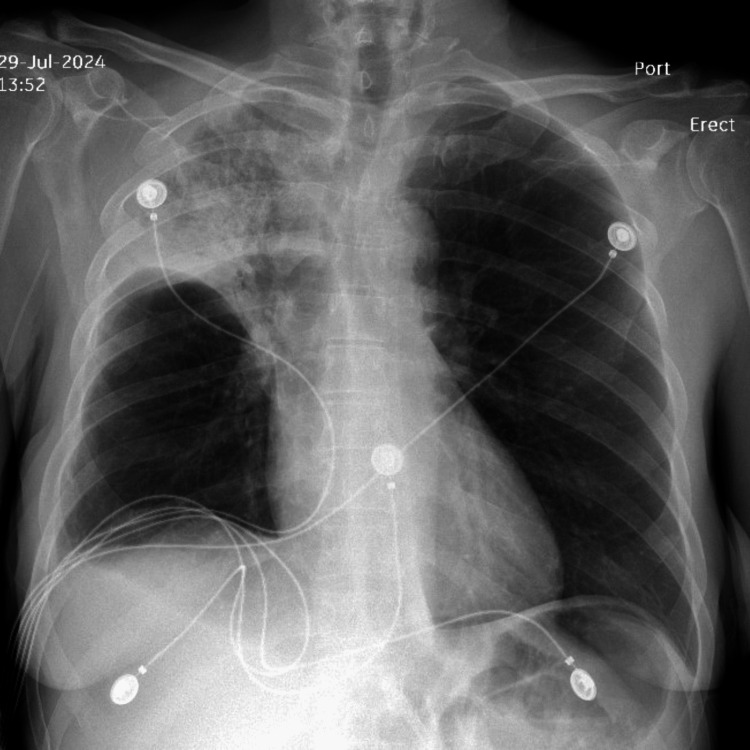
Chest radiograph obtained prior to discharge showing further resolution of right upper lobe consolidation.

The patient self-extubated on Day 2. A chest radiograph was then obtained which revealed improvement in the right upper lobe consolidation (Figure 4). Blood gasses after extubation revealed a pH of 7.30, PCO_2_ 43 tor, and PO_2_ 161 torr while breathing two liters of oxygen by nasal cannula. Arterial blood gas obtained on the evening of Day 2 before discharge revealed a pH of 7.45, PCO_2_ 33 torr, and PO_2_ 174 torr. The patient was discharged into police custody on Day 3, being ambulatory with normal oxygen saturation on room air, awake and alert without evidence of neurological sequelae, and prescribed outpatient oral antibiotics.

## Discussion

The risk of life-threatening aspiration of contraband while attempting to conceal it from law enforcement is ever-present. Most law enforcement agencies have protocols in place for dealing with the apprehended who are suspected of orally concealing contraband, which typically involves ordering the person to “spit it out” or using minimal force in an attempt to open their mouth. It should be noted that exact procedures can vary widely depending on the jurisdiction and specific circumstances [6,7]. Police, when confronted with the situation, are often left with limited options on the scene if the person is noncompliant with their commands. Since using physical force in attempts to retrieve contraband could increase the risk of aspiration, the best option left to law enforcement is to acquire assistance from emergency medical personnel. It is further believed that transport to the nearest ED for management is best if the arrestee continues to resist, as happened in the above situation.

There are reports in the literature referencing the aspiration of drug contraband in which airway obstruction was noted to be a postmortem finding [8]. One report relates the case of a 28-year-old healthy man who collapsed while being arrested by the police for alleged possession of heroin and was dead on admission to the hospital. An autopsy revealed a complete occlusion of the laryngeal opening by a plastic bag containing 24 packets of heroin powder [9]. Another report addresses the potential for aspiration: a patient was found to have a small plastic bag containing heroin in his oropharynx while an anesthesiologist was intubating the patient during operative management of a gunshot wound [10]. A remarkable case was that of a 23-year-old man who was running from police after attempting to swallow contraband. He collapsed, seized, and experienced a cardiopulmonary arrest. Cardiopulmonary resuscitation was performed in the field for 25 minutes, was intubated - which apparently pushed the contraband down the trachea as a bag of “crack” cocaine was found just above the carina. The patient underwent rigid bronchoscopy and the airway was cleared. The patient was eventually discharged from the hospital without suffering an apparent cerebral anoxic injury [11].

The Heimlich maneuver was attempted in this patient without effect. There is little doubt that the maneuver (with a success rate of 70 to 86%) has had a huge impact on the death rate from choking, with an estimated over 50,000 lives saved since its introduction [12]. It is suspected that the reported successes refer to aspirated food, and probably not plastic bags. Our patient was necessarily supine during the maneuver, which may be more effective than if applied in the standing position [13]. The maneuver was performed using high-velocity thrusts applied with both hands on each side of the chest. This method may generate higher pressures favoring dislodgement [14]. The maneuver was abandoned after two attempts due to concern for causing gastric rupture or other intraabdominal injury [15-17].

## Conclusions

This case highlights the need for high-acuity monitoring and preparedness for airway management in patients presenting with suspected drug concealment. Law enforcement particularly would be well served to evaluate existing protocols for the management of oral contraband concealment in the field.
